# Is the Conformational Ensemble of Alzheimer’s A*β*10-40 Peptide Force Field Dependent?

**DOI:** 10.1371/journal.pcbi.1005314

**Published:** 2017-01-13

**Authors:** Christopher M. Siwy, Christopher Lockhart, Dmitri K. Klimov

**Affiliations:** School of Systems Biology, George Mason University, Manassas, Virginia, United States of America; UNC Charlotte, UNITED STATES

## Abstract

By applying REMD simulations we have performed comparative analysis of the conformational ensembles of amino-truncated A*β*10-40 peptide produced with five force fields, which combine four protein parameterizations (CHARMM36, CHARMM22*, CHARMM22/cmap, and OPLS-AA) and two water models (standard and modified TIP3P). A*β*10-40 conformations were analyzed by computing secondary structure, backbone fluctuations, tertiary interactions, and radius of gyration. We have also calculated A*β*10-40 ^3^*J*_*HNHα*_-coupling and RDC constants and compared them with their experimental counterparts obtained for the full-length A*β*1-40 peptide. Our study led us to several conclusions. First, all force fields predict that A*β* adopts unfolded structure dominated by turn and random coil conformations. Second, specific TIP3P water model does not dramatically affect secondary or tertiary A*β*10-40 structure, albeit standard TIP3P model favors slightly more compact states. Third, although the secondary structures observed in CHARMM36 and CHARMM22/cmap simulations are qualitatively similar, their tertiary interactions show little consistency. Fourth, two force fields, OPLS-AA and CHARMM22* have unique features setting them apart from CHARMM36 or CHARMM22/cmap. OPLS-AA reveals moderate *β*-structure propensity coupled with extensive, but weak long-range tertiary interactions leading to A*β* collapsed conformations. CHARMM22* exhibits moderate helix propensity and generates multiple exceptionally stable long- and short-range interactions. Our investigation suggests that among all force fields CHARMM22* differs the most from CHARMM36. Fifth, the analysis of ^3^*J*_*HNHα*_-coupling and RDC constants based on CHARMM36 force field with standard TIP3P model led us to an unexpected finding that *in silico* A*β*10-40 and experimental A*β*1-40 constants are generally in better agreement than these quantities computed and measured for identical peptides, such as A*β*1-40 or A*β*1-42. This observation suggests that the differences in the conformational ensembles of A*β*10-40 and A*β*1-40 are small and the former can be used as proxy of the full-length peptide. Based on this argument, we concluded that CHARMM36 force field with standard TIP3P model produces the most accurate representation of A*β*10-40 conformational ensemble.

## Author Summary

Dependence of protein conformational ensembles on force field parameterizations limits the predictive power of molecular dynamics simulations. To address this problem, we evaluated five all-atom force fields for their consistency in reproducing the conformational ensemble of Alzheimer’s A*β*10-40 peptide. To generate conformational ensembles, we have used replica exchange molecular dynamics and computed A*β*10-40 secondary and tertiary structures. We found that, although all force fields predict A*β*10-40 unfolded structure, they strongly disagree on helix and *β* propensities and tertiary structure distributions. We have also calculated A*β*10-40 J-coupling and residual dipolar coupling constants and compared them with the experimental data for the full-length A*β*1-40 peptide. Unexpectedly, we determined that in silico A*β*10-40 and experimental A*β*1-40 constants are in better agreement than these quantities computed and measured previously for identical peptides, such as A*β*1-40 or A*β*1-42. We then concluded that the conformational ensembles of A*β*10-40 and A*β*1-40 are similar and on this basis argue that CHARMM36 force field with standard TIP3P water model provides the most accurate description of A*β*10-40. Although our objective was not to evaluate the biomolecular force fields in general, our study is expected to facilitate their proper selection for the simulations of Alzheimer’s peptides.

## Introduction

A*β* peptides linked to the development of Alzheimer’s disease (AD) are produced, through a normal cellular proteolysis, in a variety of alloforms, which differ with respect to sequence length and the extent of amino- or C-terminal truncation [1–3]. The most abundant form is a 40-residue version A*β*1-40, which constitutes about 90% of all A*β* species in cerebrospinal fluid [4]. Virtually all A*β* peptides are highly amyloidogenic [5, 6] and play a central role in amyloid cascade hypothesis, which explains AD pathogenesis on the basis of multi-stage aggregation of A*β* species. In this process, A*β* monomers represent initial species involved in spontaneous aggregation. Moreover, according to experimental studies fibril elongation is also largely driven by deposition of A*β* monomers to the fibril edges [7]. Generally, A*β* peptides display a high level of cytotoxicity [2, 8–10], which is related to their ability to readily bind to cellular lipid bilayers and disrupt their structure [11]. Although the mechanism of binding to lipid bilayers is likely to be concentration dependent, A*β* peptides predominantly bind as monomers rather than oligomers at nanomolar concentrations [12, 13].

A*β* peptides belong to the class of intrinsically disordered proteins implying that they lack well defined native structure in aqueous environment. Indeed, experimental investigations, including solution NMR studies, have shown that the conformational ensemble of A*β* monomer in water is populated by heterogeneous coil-like conformations [14–17]. More recently, several NMR measurements, including chemical shifts, nuclear Overhauser effects, and *J*-couplings, have been used to confirm that A*β* peptides adopt generally random coil structures at neutral pH [18]. At the same time, careful analysis of electron paramagnetic resonance studies revealed that A*β* monomers still contain short structured regions (His14-Val18, Gly29-Ala30, and Gly38-Val40) under normal physiological conditions [19]. Similarly, several NMR studies have pointed to a formation of a turn or bend structures in the sequence region (Phe20-Ser26) between the central hydrophobic cluster (Leu17-Ala21) and the C-terminal (Ala30-Val40) [15, 17]. Due to generally disordered state of A*β* in water, it is not surprising that A*β* conformations are highly dependent on solvent properties. For example, in the membrane-like environments A*β* conformational ensemble undergoes considerable reorganization manifested in the formation of helical structure in the sequence regions Glu15-Val24 and Gly29-Met35 [20–22]. Moreover, A*β* helix propensity is pH dependent illustrated by the observation that the Glu15-Val24 helix, but not the C-terminal helix, becomes destabilized at normal pH. Conformational plasticity of A*β* peptides is also consistent with mutagenesis studies. For example, Iowa (D23N) or Osaka deletion (E22Δ) mutants aggregate significantly faster than the wild-type [23, 24]. Similarly, according to *in vitro* studies many single-point mutations mainly affecting hydrophobic or charged residues can either accelerate or reduce A*β* aggregation propensity [25, 26]. One may expect that A*β* monomeric conformations, being the initial species involved in aggregation, are impacted by these single-point mutations.

A survey of experimental findings presented above suggests that computational characterization of the conformational ensemble formed by A*β* monomers is important for understanding its aggregation and cytotoxicity. Several previous molecular dynamics studies have probed A*β* monomers in water. Garcia and coworkers have studied the conformations of A*β*1-40 and A*β*1-42 peptides using replica exchange molecular dynamics (REMD) simulations extended to microsecond timescales [16, 17, 27]. Consistent with the experiments, their analysis revealed generally disordered A*β* conformational ensemble augmented by several structured regions, especially in the C-terminal of A*β*1-42, where a *β*-hairpin has been detected. Qualitatively similar conclusions have been reached in the recent REMD study conducted by Head-Gordon and coworkers [28]. In our previous studies, we have utilized REMD and all-atom CHARMM22 force field with CMAP corrections to investigate the conformations of amino-truncated A*β*10-40 peptide in water [29]. We found that, similar to the full-length peptide, A*β*10-40 samples predominantly turn and random coil structures, whereas helical and especially *β*-sheet propensities are low. Furthermore, the peptide almost completely lacks tertiary structure with most stable intrapeptide interactions forming between the amino acids adjacent along the sequence. In light of disordered state of A*β* monomer it is important to test the dependence of its conformational ensemble on the force fields employed in the simulations. Recently, such investigation has been carried out for A*β*1-40 monomer, which was probed using OPLS-AA/L, AMBERff99sb-ILDN, and CHARMM22* protein force fields and several water models [30]. All the three force fields predicted the formation of *β*-structure in the Leu17-Ala21 and Ala30-Leu34 regions, but with markedly different *β* propensities. In particular, OPLS-AA/L and AMBERff99sb-ILDN simulations revealed stable *β*-structures, whereas suppressed *β* fraction has been observed in the CHARMM22* force field. It is conceivable that the three force fields overestimate the *β* propensity in A*β* monomer, which is expected to be low according to the NMR studies [18]. Indeed, REMD study of two natively unfolded peptides, NTL9 (1–22) and NTL9 (6–17), using AMBERff99sb-ILDN, CHARMM22/CMAP, and CHARMM36 force fields [31] has shown that both CHARMM force fields predict much smaller *β* fraction than AMBERff99sb-ILDN. Additionally, the parameterization of water may affect peptide conformational ensembles. This point has been recently demonstrated using REMD and CHARMM36 for two Ala-rich peptides and GB1 peptide, which form considerably more solvated and extended structures with modified TIP3P water model compared to its standard version [32].

Because previous investigations have emphasized the importance of force field parameterization, a natural question arises about how a force field affects sampling of amino-truncated A*β*10-40 peptide, which was extensively studied by us in the context of peptide-lipid bilayer interactions [33–36]. To address this question, we used all-atom REMD simulations and performed a systematic comparison of A*β*10-40 conformational ensembles in four protein force fields (CHARMM36, CHARMM22*, CHARMM22/CMAP, OPLS-AA) and two water models (standard and modified TIP3P). We show that although all force fields are consistent in predicting the A*β* propensity to form turn and random coil structures, they strongly disagree on the extent and distribution of tertiary interactions or helix and *β* propensities in A*β* monomer. We have also compared the *J*-coupling and residual dipolar coupling (RDC) constants computed from A*β*10-40 simulations with A*β*1-40 experimental data. Surprisingly, we found that *in silico* A*β*10-40 conformational ensemble produced by CHARMM36 force field agrees better with the experimental measurements than *in silico* A*β*1-40 ensemble simulated earlier [17, 27]. Based on these comparisons we suggest that A*β*10-40 can serve as a proxy of the full-length A*β*1-40 peptide and the CHARMM36 force field with standard TIP3P water model provides most accurate reproduction of A*β* conformational ensemble.

## Materials and Methods

### Molecular model and simulations

**Fig 1:**
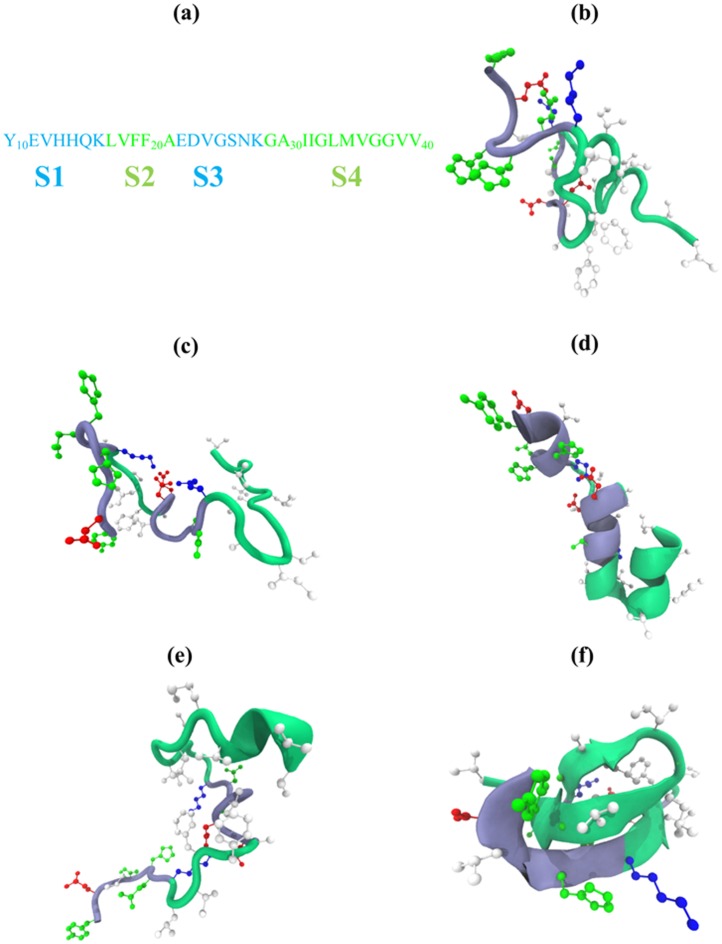
A*β*10-40 sequence and conformations in different force fields. (a) A*β*10-40 sequence is divided in four regions: hydrophilic N-terminal (S1, residues 10-16), central hydrophobic cluster (S2, residues 17-21), hydrophilic turn (S3, residues 22-28), and hydrophobic C-terminal (S4, residues 29-40). (b-f) Representative conformations of A*β*10-40 peptide in five force fields: (b) C36, (c) C36s, (d) C22*, (e) C22cmap, and (f) OPLS-AA. C36, C36s, and C22cmap structures in (b), (c), (e) show disordered peptide, whereas C22* in (d) and OPLS-AA in (f) structures illustrate helix or *β* propensities characterizing the respective force field. Side chains are colored to represent hydrophobic (in light grey), polar (in green), positively charged (in blue), and negatively charged (in red) residues. The backbone coloring follows the scheme used in (a).

To explore the impact of force field parameterizations, we have performed molecular dynamics (MD) simulations of A*β*10-40 peptide, which is an amino-truncated fragment of the full-length peptide A*β*1-40 (Fig 1a). In all, we have investigated four all-atom protein force fields and two explicit water models [37, 38] resulting in five simulation systems, which utilized CHARMM36 [39] with modified TIP3P water model (denoted as C36), CHARMM36 with standard TIP3P water model (C36s), CHARMM22* with modified TIP3P water model (C22*) [40], CHARMM22 with CMAP corrections and modified TIP3P water model (C22cmap) [41], and OPLS-AA with modified TIP3P water model (OPLS-AA) [42]. The C22cmap system was already studied by us previously [29, 43, 44] and is used here to expand the force fields comparison. All simulation systems contained a single A*β*10-40 peptide, 4959 water molecules, and one sodium ion to set the net system charge to zero. In all, the simulation systems contained 15,354 atoms. A*β* termini were capped with acetylated and aminated groups. The charged states of amino acids corresponded to neutral pH (in particular, histidines were deprotonated). For all force fields we used periodic boundary conditions with the cubic unit cell having the edge dimension of about 53.8 Å and resulting in the mass density of 0.9848 *g*/*cm*^3^ at 330 *K*. Non-bonded interactions were computed using a smooth switching functions acting within the interval of 8 and 12 Å. Electrostatic interactions were computed using particle mesh Ewald summation with the grid size of ≈ 1Å. All hydrogen associated covalent bonds except in water molecules were treated as rigid by applying ShakeH algorithm. Water molecules were treated as rigid using SETTLE algorithm. All simulations used the integration step of 1 *fs*. Full electrostatic evaluation frequency was set to 4 integration steps.

### Replica exchange protocol

To produce exhaustive sampling of A*β*10-40 conformational ensembles we have utilized canonical (NVT) replica exchange molecular dynamics (REMD) [45], which is implemented in NAMD MD program [46]. For all systems we used *R* = 40 replicas distributed exponentially in the temperature range from 300 to 440 *K*. Canonical ensembles in the replicas were generated by applying underdamped Langevin dynamics with the damping coefficient *γ* = 5 *ps*^−1^. Replica exchanges were attempted every 2 *ps* between all neighboring replicas along a temperature scale resulting in the average acceptance rates ranging between 27 and 29%. For each simulation system we have produced four REMD trajectories collecting in total 3.2 *μs* of sampling (or 80 *ns* per replica). Each REMD trajectory has been initiated with random initial conformations equilibrated at 330 *K* with preliminary isothermal-isobaric simulations to set correct mass densities. In addition, each replica was equilibrated at its own temperature for an additional 1 *ns*. With this approach no sampling data from REMD trajectories needed to be discarded as non-equilibrated. Although probing REMD convergence is generally a difficult task (see, e.g., [47]), our analysis in S1 Text suggests that the resulting simulation times appear sufficient for sampling convergence.

### Computation of structural probes

Secondary structures in A*β* were assigned using the STRIDE program [48]. A helical state includes *α*-, 3_10_-, or *π*-helix conformations, whereas a *β*-strand state includes extended conformations or isolated bridges. Tertiary interactions were probed by side chain contacts. A contact occurs if the distance between the geometric centers of heavy atoms in two side chains is less than 6.5 Å. This cutoff approximately corresponds to the onset of hydration of side chains as their separation increases. We classified the contacts between residues *i* and *j* as long-range if |*j* − *i*| ≥ 5 or short-range otherwise. To explore the fluctuations in peptide backbone, we computed the standard deviations *δϕ*(*i*) and *δψ*(*i*) for backbone dihedral angles *ϕ* and *ψ* of a residue *i*. These standard deviations are referred to as root-mean-square fluctuations (RMSF). To evaluate peptide dimensions we computed the radius of gyration, *R*_*g*_, using the positions of side chain centers of mass and C_*α*_ atoms.

To quantitatively evaluate the consistency between the experimental and computational conformational ensembles we have utilized two quantities. The first is the ^3^*J*_*HNHα*_ coupling constants associated with three-bond coupling interaction between *H*_*N*_ and *H*_*α*_ protons. These constants are sensitive to peptide’s secondary structure [49]. In this study we used three sets of experimentally measured *J*-coupling constants, *J*_*exp*_, for A*β*1-40 peptide [16–18]. *In silico* *J*-coupling constants, *J*_*comp*_, were determined from the backbone dihedral angles using Karplus equation [50]

$$J_{comp}=A\cdot cos^{2}\left(\phi -60^{{}^{\circ}}\right)+B\cdot cos\left(\phi -60^{{}^{\circ}}\right)+C,$$ (1)

where *ϕ* is a backbone dihedral angle and *A*, *B*, and *C* are the coefficients determined by fitting with the experimental data. We used three sets of coefficients reported by Pardi *et al* (*A* = 6.4, *B* = −1.4, and *C* = 1.9) [51], Brueschweiler *et al* (*A* = 9.5, *B* = −1.4, and *C* = 0.3) [52], and Vuister *et al* (*A* = 6.51, *B* = −1.76, and *C* = 1.60) [53]. The N-terminal amino acid was excluded from our computation of *J*-couplings as its *ϕ* angle may be distorted by the capping group.

As a second quantity we chose residual dipolar coupling (RDC) constants experimentally measured for A*β*1-40 peptide [54]. RDC measurements probe orientation of amide *NH* bonds in protein backbones with respect to external magnetic field, and can identify long-range structural correlations across biomolecular structure. To compute RDC constants from *in silico* structures we used the Prediction of ALignmEnt from Structure (PALES) program [55]. We processed our simulation structures by using default PALES settings, including the application of steric interaction model, which determines alignment orientation based on steric properties of a molecule. As described in S1 Text we applied global alignment of A*β*10-40 structures for RDC computations. Once the RDC constants were produced, they were multiplied by the scaling factors determined from the least-squares fitting that minimizes the deviation between the experimental and *in silico* data.

To assess the similarity between experimental and computational quantities, we used the Pearson’s correlation coefficient (PCC), root mean square deviations (RMSD), and quality factor *Q* defined as $Q=\sqrt{\sum_{k}{\left(D_{comp,k}-D_{exp,k}\right)}^{2}}/\sqrt{\sum_{k}D_{exp,k}^{2}}$, where *D*_*exp*,*k*_ and *D*_*comp*,*k*_ are the experimental measurements and their computed counterparts available for amino acids *k*. The sum is taken over all amino acids for which experimental and computed values are simultaneously available. We applied *Q* to evaluate agreement for *J*-coupling or RDC constants. To preserve consistency with our previous studies all ensemble averages were computed using weighted histogram analysis method (WHAM) [56] of REMD data at 330K. *J*-coupling and RDC constants were computed at 300K, which is the closest simulation temperature to experimental conditions (≈ 280K) [16–18].

## Results

### Conformational ensemble of A*β*10-40 monomer in CHARMM36 force field with modified TIP3P water model

**Fig 2:**
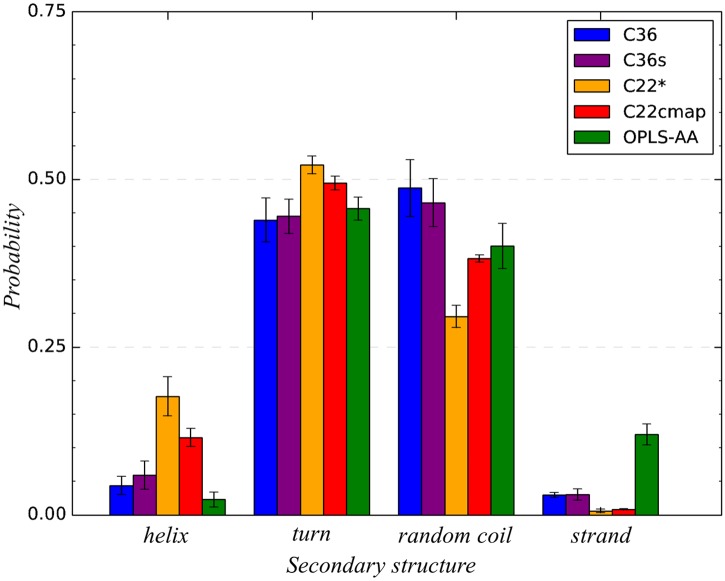
A*β*10-40 secondary structure in different force fields. Total fractions of helix, turn, random coil, and *β* secondary structures for each of the five force fields probed in REMD simulations. All force fields predict dominance of turn and random coil conformations. C22* and OPLS-AA reveal moderate helix and *β*-structure propensities, respectively.

**Fig 3:**
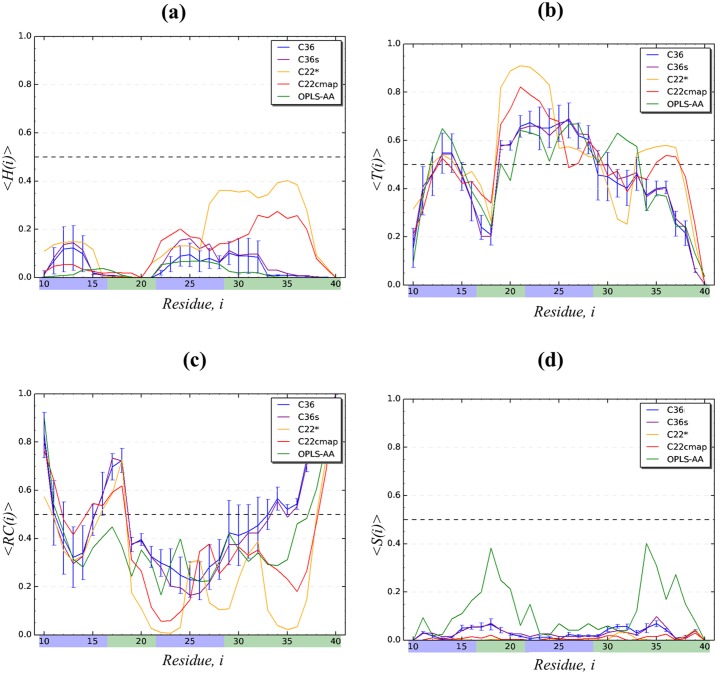
Residue-specific A*β*10-40 secondary structure in different force fields. Distributions of secondary structure in A*β*10-40 peptide with respect to sequence positions *i* for five force fields: (a) helix propensities 〈*H*(*i*)〉; (b) turn propensities 〈*T*(*i*)〉; (c) random coil propensities 〈*RC*(*i*)〉; (d) *β* propensities 〈*S*(*i*)〉. For clarity, sampling errors represented by vertical bars are shown for the C36 simulations only. Sequence regions are identified by the color scheme used in Fig 1a. C22* force field displays a significant helix structure in S3 and S4 regions, and the OPLS-AA system has a propensity for *β*-structure in S2 and S4.

**Fig 4:**
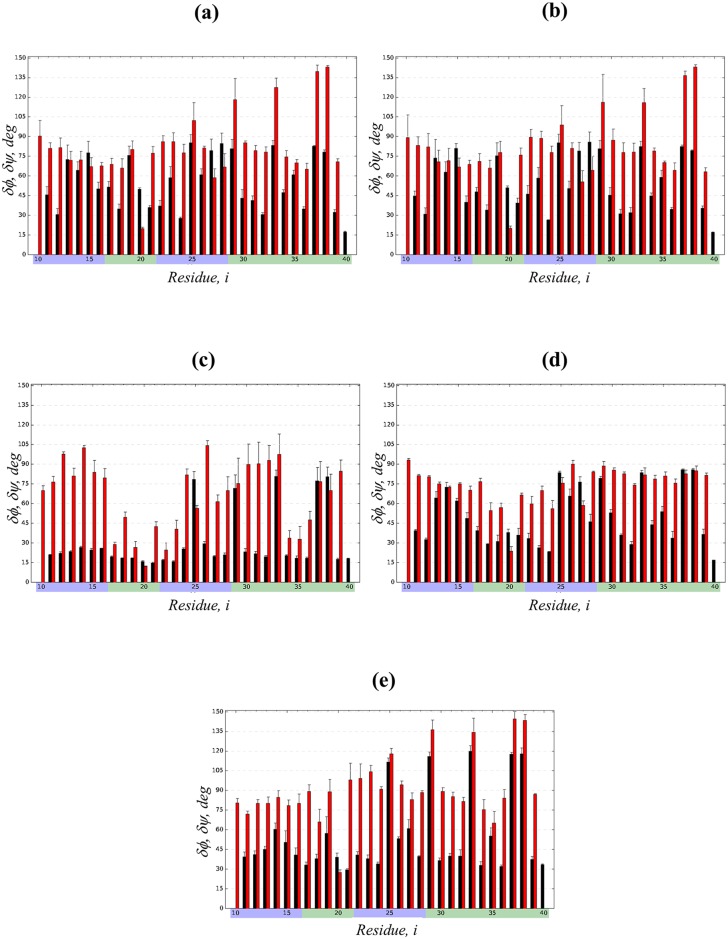
A*β* backbone fluctuations in different force fields. Root-mean-square fluctuations (RMSF), *δϕ*(*i*) (black bars) and *δψ*(*i*) (red bars), in the backbone dihedral angles *ϕ* and *ψ* for A*β*10-40 amino acids *i* computed in five force fields: (a) C36, (b) C36s, (c) C22*, (d) C22cmap, and (e) OPLS-AA. Sampling errors are shown by vertical bars. Sequence regions are identified by the color scheme used in Fig 1a. The plots show that out of all force fields C22* predicts the most rigid backbone, especially in S2 and S4 regions.

We begin the comparison of different force fields with the detailed analysis of the conformational ensemble of A*β*10-40 monomer in CHARMM36 force field with modified TIP3P water model (denoted as C36). We first focus on the A*β*10-40 secondary structure. It follows from Fig 2 and Table 1 that its conformational ensemble is dominated by random coil (〈*RC*〉 = 0.49 ± 0.04) and turn (〈*T*〉 = 0.44 ± 0.03), which together account for 93% of all amino acid states. Indeed, the turn structure is stable (i.e., its fraction 〈*T*(*i*)〉 > 0.5) for few S1 residues (His13, His14) and within the long sequence span Phe19-Gly29 covering the part of hydrophobic region S2, the entire hydrophilic S3, and the beginning of C-terminal S4. Random coil structure dominates the N- and C-terminals and the Gln15-Val18 region. The occurrence of helix and *β*-structure is negligible (≤0.04). In fact, Fig 3 shows that the helical propensity 〈*H*(*i*)〉 is weak throughout A*β*10-40 sequence being always ≲ 0.1. To explore the structural fluctuations in A*β*10-40 backbone we have computed the root-mean-square fluctuations (RMSF), *δϕ*(*i*) and *δψ*(*i*), in backbone dihedral angles *ϕ* and *ψ* of amino acids *i*. Fig 4a shows that apart from enhanced fluctuations of Gly *ψ* angles the distributions *δϕ*(*i*) and *δψ*(*i*) are fairly uniform throughout A*β* sequence. The most rigid backbone conformation is observed for hydrophobic Phe20. The values of *δϕ* and *δψ* averaged over the entire A*β*10-40 sequence are 55.2 ± 1.6° and 81.9 ± 3.8°, respectively. Relatively uniform distribution of the backbone fluctuations in A*β* sequence is consistent with the lack of well-defined secondary structure, such as helices or *β*-strands.

**Table 1:** Secondary Structure in A*β*10-40 Peptide.

Force Field	Water Model	〈*H*〉^a^	〈*T*〉^b^	〈*RC*〉^c^	〈*S*〉^d^
CHARMM36	mTIP3P	0.04 ± 0.01	0.44 ± 0.03	0.49 ± 0.04	0.03 ± 0.00
CHARMM36	sTIP3P	0.06 ± 0.02	0.45 ± 0.03	0.47 ± 0.04	0.03 ± 0.01
CHARMM22*	mTIP3P	0.18 ± 0.03	0.52 ± 0.01	0.30 ± 0.02	0.01 ± 0.00
CHARMM22/cmap^e^	mTIP3P	0.12 ± 0.01	0.49 ± 0.01	0.38 ± 0.01	0.01 ± 0.00
OPLS-AA	mTIP3P	0.02 ± 0.01	0.46 ± 0.02	0.40 ± 0.03	0.12 ± 0.02

^*a*^Fraction of helix.

^*b*^Fraction of turn.

^*c*^Fraction of random coil.

^*d*^Fraction of *β*-sheet.

^*e*^data from [29, 43]

**Fig 5:**
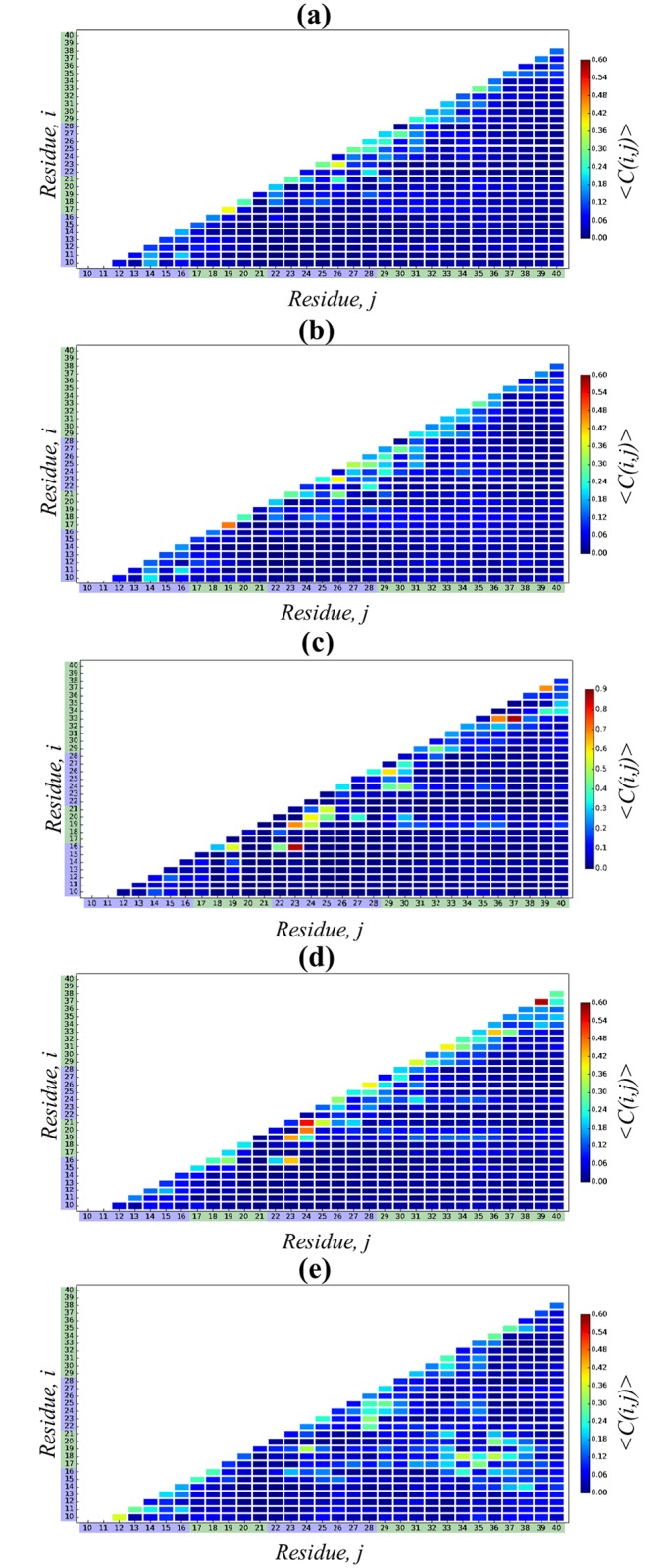
A*β*10-40 tertiary interactions in different force fields. Intrapeptide contact maps, 〈*C*(*i*, *j*)〉, present the probabilities of forming contacts between residues *i* and *j* in A*β*10-40 peptide for five force fields: (a) C36, (b) C36s, (c) C22*, (d) C22cmap [44], and (e) OPLS-AA. The bars on the right-side color code 〈*C*(*i*, *j*)〉 values. Sequence regions are identified by the color scheme used in Fig 1a. C36, C36s, and C22cmap contact maps reveal lack of stable long-range interactions, whereas C22* displays few exceptionally strong long- and short-range contacts. OPLS-AA contact map is characterized by extensive but flickering tertiary interactions.

We next investigate the A*β*10-40 tertiary structure by probing the formation of intrapeptide interactions. Fig 5a presents the peptide contact map 〈*C*(*i*, *j*)〉, which visualizes the probabilities of forming contacts between amino acid side chains. This figure suggests that there are very few stable interactions in A*β* peptide, i.e., those occurring with the probability 〈*C*(*i*, *j*)〉 > 0.35. According to Table 2 there are no stable long-range (|*i* − *j*| ≥ 5) contacts, and there are only two stable short-range (|*i* − *j*| < 5) contacts, namely, Leu17-Phe19 and Asp23-Ser26. The Leu17-Phe19 contact is likely to explain a rigid backbone conformation at Phe20. The probability of forming a salt bridge Asp23-Lys28, which is important for amyloid fibril assembly, is low being equal to 〈*C*(23, 28)〉 = 0.10 ± 0.03. However, weak electrostatic interactions are formed between Glu22 and Lys28 (0.21 ± 0.13) and between Glu11 and Lys16 (0.20 ± 0.11). Overall, the average number of all side chain contacts forming in A*β* monomer is 〈*C*〉 = 21.1 ± 1.4, of which 10.7 ± 0.9 (or 51%) are long-range.

**Table 2:** Five top side chain contacts in A*β*10-40 peptide for C36 force field.

long-range			short-range		
Rank	contact	〈*C*(*i*, *j*)〉	Rank	contact	〈*C*(*i*, *j*)〉
1	21–26	0.25 ± 0.13	1	17–19	0.39 ± 0.11
2	22–28	0.21 ± 0.13	2	23–26	0.38 ± 0.07
3	11–16	0.20 ± 0.11	3	23–25	0.29 ± 0.10
4	24–29	0.17 ± 0.09	4	25–27	0.29 ± 0.11
5	26–31	0.15 ± 0.12	5	33–35	0.29 ± 0.03

**Fig 6:**
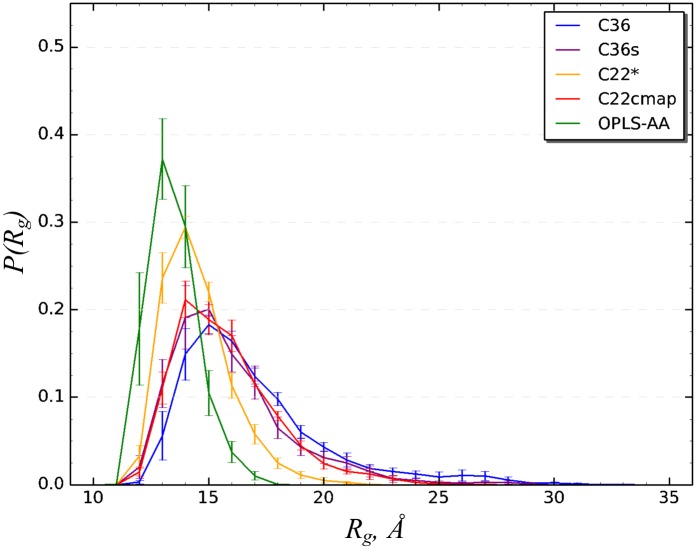
A*β* radius of gyration in different force fields. Probability distributions, *P*(*R*_*g*_), for the radius of gyration *R*_*g*_ of A*β*10-40 peptide computed for five force fields. Vertical bars represent sampling errors. The plots show that, in contrast to C36, C36s, and C22cmap force fields, C22* and OPLS-AA predict collapsed peptide structures.

Finally, we consider the probability distribution *P*(*R*_*g*_) of A*β*10-40 radius of gyration *R*_*g*_. Fig 6 shows that for C36 *P*(*R*_*g*_) is broad with the maximum at *R*_*g*_ ≈ 15 Å. The equilibrium value of *R*_*g*_, 〈*R*_*g*_〉, is 16.9 ± 0.5 Å, which is the largest among all force field considered (see below). In addition, we have computed the average end-to-end distance 〈*R*_1*N*_〉 = 24.6 ± 1.2Å, which is also the largest among other force fields. Thus, A*β*10-40 peptide in C36 force field lacks stable secondary or tertiary structure and forms expanded conformations as illustrated in Fig 1b.

### Conformational ensemble of A*β*10-40 monomer in CHARMM36 force field with standard TIP3P water model

To check the impact of water model, we repeated CHARMM36 REMD simulations of A*β*10-40 peptide using standard TIP3P water (denoted as C36s). Following the analysis for C36 we have computed A*β* secondary and tertiary structure. Fig 2 and Table 1 demonstrate that in close agreement to C36 simulations A*β*10-40 primarily samples random coil (〈*RC*〉 = 0.47 ± 0.04) or turn (〈*T*〉 = 0.45 ± 0.03) conformations, which together represent 92% of all residue states. In contrast, helix or *β*-state occur rarely. Fig 3 further reveals that the residue-specific turn 〈*T*(*i*)〉 and helix 〈*H*(*i*)〉 propensities are almost identical to those observed in C36. Similar to C36, stable turn structure is present at His13-His14 and in the region Phe19-Gly29. Consequently, the root-mean-square deviations (RMSD) between 〈*T*(*i*)〉 and helix 〈*H*(*i*)〉 distributions computed from C36s and C36 simulations are low (0.02 and 0.03). Random coil distributions between C36s and C36 also nearly match. Furthermore, according to Fig 4a and 4b a very close agreement is observed in the RMSF distributions, *δϕ*(*i*) and *δψ*(*i*), as confirmed by the low respective RMSD values (3.9 and 3.2°). As expected the average values 〈*δϕ*〉 and 〈*δψ*〉 computed using all amino acids are 54.6 ± 1.9° and 81.1 ± 5.0°, which are nearly identical to those of C36.

To analyze A*β* tertiary structure we have computed the contact map 〈*C*(*i*, *j*)〉, which is presented in Fig 5b. In general, C36s tertiary interactions are very similar to those seen for C36. It follows from Table 3 that A*β* peptide has no stable long-range contacts and the same two stable short-range contacts as in C36 are formed. There are four common contacts among top five long-range interactions in C36s and C36 simulations (Ala21-Ser26, Glu22-Lys28, Glu11-Lys16, Val24-Gly29), whereas three short-range contacts are shared between the two simulations (Leu17-Phe19, Asp23-Ser26, Gly25-Asn27). As in C36 the probability of forming the salt bridge Asp23-Lys28 is low (0.14 ± 0.07). Overall, the RMSD computed between C36 and C36 contact maps is small (0.02) confirming their similarity. It is noteworthy, however, that in C36s simulations the peptide forms, on an average, more side chain contacts than in C36, as their number reaches 〈*C*〉 = 23.5 ± 1.4, of which 12.8 ± 0.8 (or 54%) are classified as long-range. Furthermore, comparison of the probability distributions *P*(*R*_*g*_) for A*β*10-40 radius of gyration in Fig 6 reveals that the C36s distribution is systematically shifted to smaller *R*_*g*_ values suggesting that the C36s peptide is more compact. Indeed, from Fig 6 we determine that the equilibrium 〈*R*_*g*_〉 is 15.9 ± 0.4Å, which is smaller than the C36 value. Thus, although the secondary and tertiary structure propensities in C36s and C36 simulations are in close agreement, the former generates more compact structures with larger number of intrapeptide interactions (Fig 1c).

**Table 3:** Five top side chain contacts in A*β*10-40 peptide for C36s force field.

long-range			short-range		
Rank	contact	〈*C*(*i*, *j*)〉	Rank	contact	〈*C*(*i*, *j*)〉
1	21–26	0.30 ± 0.14	1	17–19	0.47 ± 0.12
2	22–28	0.23 ± 0.17	2	23–26	0.39 ± 0.06
3	24–29	0.23 ± 0.13	3	25–27	0.33 ± 0.15
4	11–16	0.21 ± 0.14	4	25–28	0.30 ± 0.11
5	25–31	0.18 ± 0.10	5	27–30	0.28 ± 0.18

### Conformational ensemble of A*β*10-40 monomer in CHARMM22* force field

CHARMM22* (C22*) is another version of CHARMM force field, which was developed to address conformational biases in CHARMM22cmap [40]. We performed REMD simulations of A*β*10-40 monomer using C22* and studied its conformational ensemble. As demonstrated by Fig 2 and Table 1 the peptide forms predominantly turn conformations (〈*T*〉 = 0.52 ± 0.01), whereas the fraction of random coil (〈*RC*〉 = 0.30 ± 0.02), while still significant, is reduced compared to C36 and C36s. A distinctive feature of C22* simulations is an elevated fraction of helical structure, which reaches 0.18 ± 0.03 (a more than four-fold increase compared to C36). Occurrence of *β*-states is negligible. Fig 3 further underscores differences in the secondary structure distributions at a residue level. The C22* turn fraction 〈*T*(*i*)〉 is noticeably higher than the C36 turn propensities within hydrophobic S2 and hydrophilic S3 regions (Phe19-Gly25), where it exceeds 0.8. Random coil occurs near the peptide terminals and within Lys16-Val18. More importantly, C22* leads to formation of a marginally stable helix structure, particularly, in the hydrophobic C-terminal (S4 region). Indeed, according to Fig 3a within the sequence interval Asn27-Val36 the helix fraction 〈*H*(*i*)〉 approaches 0.4 in sharp contrast to C36 propensities. Consequently, the differences between turn and helix distributions observed in C22* and C36 simulations are reflected in high RMSD values, which are 0.16 and 0.19, respectively.

The RMSF distributions, *δϕ*(*i*) and *δψ*(*i*), shown in Fig 4c display distinctive features characteristic of C22* force field. Specifically, the plot reveals two sequence regions with suppressed backbone fluctuations, Val17-Asp23 and Leu34-Val36, which approximately coincide with the formation of stable turn and marginally stable helix structures. Furthermore, in C22* simulations for most sequence positions *δϕ*(*i*) is much smaller than in C36. Overall, the average RMSF 〈*δϕ*〉 and 〈*δψ*〉 observed in C22* are 30.2 ± 1.5° and 66.1 ± 4.6°, i.e., compared to C36 a particularly significant decrease by a factor of 1.8 is seen in 〈*δϕ*〉. It is then not surprising that the RMSD for *δϕ*(*i*) and *δψ*(*i*) distributions computed between C22* and C36 simulations are 31.1° and 33.0°, respectively, which are about an order of magnitude larger than those comparing C36 force fields.

The C22* equilibrium contact map 〈*C*(*i*, *j*)〉 displayed in Fig 5c shows a formation of multiple side chain contacts, of which eight long-range and ten short-range contacts are stable. Moreover, Table 4 lists top five long-range contacts, one of which, the salt bridge Lys16-Asp23, is formed with exceptionally high probability of 0.85 ± 0.02. Two hydrophobic long-range contacts, Phe19-Val24 and Val24-Ala30, also occur with high probabilities (0.52 ± 0.04 and 0.46 ± 0.08). Interestingly, in C22* simulations Asp23-Lys28 salt bridge is effectively disrupted (0.06 ± 0.03). Also, multiple very stable short-range contacts are observed, such as the helix-like contact Gly33-Gly37 (0.86 ± 0.03). Overall, the equilibrium number of side chain contacts in A*β*10-40 is 〈*C*〉 = 29.0 ± 0.8, of which 〈*C*_*LR*_〉 = 15.4 ± 0.3 (or 53%) are long-range. Compared to C36, the values of 〈*C*〉 and 〈*C*_*LR*_〉 are larger by 37% and 44%, respectively. Finally, we note that none of the top five C22* long- or short contacts are observed in C36 or C36s simulations. Consequently, the RMSD value measuring the difference between C36 and C22* contact maps is much larger (0.12) than the RMSD comparing C36 and C36s force fields.

**Table 4:** Five top side chain contacts in A*β*10-40 peptide for C22* force field.

long-range			short-range		
Rank	contact	〈*C*(*i*, *j*)〉	Rank	contact	〈*C*(*i*, *j*)〉
1	16–23	0.85 ± 0.02	1	33–37	0.86 ± 0.03
2	19–24	0.52 ± 0.04	2	33–36	0.69 ± 0.05
3	24–30	0.46 ± 0.08	3	37–39	0.68 ± 0.07
4	16–22	0.44 ± 0.03	4	19–23	0.68 ± 0.03
5	20–25	0.44 ± 0.10	5	26–29	0.60 ± 0.05

As seen in Fig 6 the probability distribution *P*(*R*_*g*_) for A*β*10-40 monomer computed from C22* simulations peaks at smaller values of *R*_*g*_ and is more narrow than for either of C36 force fields. From Fig 6 we find 〈*R*_*g*_〉 = 14.5 ± 0.2Å, which is smaller than the respective values for C36 simulations (16.9 or 15.9 Å). The same conclusion applies to the end-to-end distance (〈*R*_1*N*_〉 = 20.0 ± 0.9 Å). In summary, compared to C36 simulations, C22* force field significantly enhances turn and, particularly, helical propensities, makes several sections of A*β* backbone rigid, and dramatically strengthens tertiary interactions as illustrated in Fig 1d resulting in peptide compaction. Therefore, there is little consistency between the conformational ensembles mapped using C22* and C36 force fields.

### Conformational ensemble of A*β*10-40 monomer in CHARMM22 force field with CMAP corrections

In our previous work [29, 43], we have used REMD to study the conformational ensemble of the A*β*10-40 monomer using CHARMM22 force field with CMAP corrections (denoted as C22cmap). Below we extend our previous C22cmap analysis to provide comparison with other force fields. We begin by focusing on A*β* secondary structure. Fig 2 and Table 1 demonstrate that in C22cmap A*β* mainly forms turn (〈*T*〉 = 0.49 ± 0.01) and random coil (〈*RC*〉 = 0.38 ± 0.01) structures. These observations are qualitatively consistent with other force fields. However, compared to both C36 the population of helical structure in C22cmap (〈*H*〉 = 0.12 ± 0.01) is elevated, whereas the formation of *β* structure is still rare. Fig 3 demonstrates residue-specific secondary structure propensities for C22cmap. Stable turn structure is observed in His13, Phe19-Gly25, Asn27-Gly29, and Met35-Gly37, which with the exception of the last region match well the C36 turn distribution. Random coil, 〈*RC*(*i*)〉, is observed in Tyr10-Glu11, Gln15-Val18, and Val39-Val40, i.e., it follows the respective C36 propensities. Fig 3a also reveals an appearance of marginally stable helix in the C-terminal region S4 (Ile32-Val36), which approximately coincides with the distribution of C22* helix. The RMSD values comparing C22cmap and C36 distributions of turn and helix structure are 0.11 and 0.12, respectively, implicating moderate differences between the two systems.

Fig 4d demonstrates that the fluctuations of backbone dihedral angles, *δϕ*(*i*) and *δψ*(*i*), with the exception for Gly residues, agree generally well with those computed for C36. However, in contrast to C36 distributions, the backbone fluctuations are suppressed in a wider interval of Val18-Val24 compared to a single position Phe20 in C36. The averages 〈*δϕ*〉 and 〈*δψ*〉 are 49.5 ± 1.0° and 74.0 ± 1.2°, which are somewhat smaller than the respective averages computed from C36 simulations. The RMSD values comparing *δϕ*(*i*) and *δψ*(*i*) between C22cmap and C36 are 13.7° and 21.1°, respectively. These RMSD values are much larger than those comparing C36 and C36s force fields, but significantly smaller (by 2.3 or 1.6 times, respectively) than the RMSD probing the difference between C36 and C22*. In line with these observations, the C22cmap fluctuations in Fig 4d, particularly *δϕ*(*i*), are generally higher than those observed for C22*.

Fig 5d presents the equilibrium contact map 〈*C*(*i*, *j*)〉 computed using C22cmap simulations. In all, we have detected the formation of nine short-range and one long-range (Lys16-Asp23) stable contacts that stands in contrast to C36 results, which showed only two stable short-range contacts. The Asp23-Lys28 salt-bridge, which is disrupted in C22* or C36, has also low probability of occurrence (0.15 ± 0.03). Moreover, Table 5 demonstrates none of the top long- or short-range contacts formed in C22cmap force field are observed in any of C36 simulations. The overall number of side chain contacts formed in C22cmap is 〈*C*〉 = 26.2 ± 0.2, of which 〈*C*_*LR*_〉 = 12.3 ± 0.2 (or 47%) are long-range. Compared to C36 simulations, 〈*C*〉 and 〈*C*_*LR*_〉 are larger by 24 and 15%, respectively. As a result, the RMSD comparing the C22 and C36 contact maps is moderately large (0.07), exceeding the RMSD between C36 and C36s simulations (0.02), but still being smaller than the RMSD comparing C36 and C22* (0.12).

**Table 5:** Five top side chain contacts in A*β*10-40 peptide for C22cmap force field^a^.

long-range			short-range		
Rank	contact	〈*C*(*i*, *j*)〉	Rank	contact	〈*C*(*i*, *j*)〉
1	16–23	0.42 ± 0.02	1	37–39	0.57 ± 0.02
2	19–24	0.23 ± 0.04	2	21–24	0.54 ± 0.03
3	24–31	0.21 ± 0.02	3	20–24	0.47 ± 0.04
4	21–27	0.20 ± 0.03	4	19–23	0.45 ± 0.04
5	16–22	0.20 ± 0.01	5	33–36	0.42 ± 0.05

^*a*^ data from [29, 43]

Last, we examined the probability distribution *P*(*R*_*g*_) plotted in Fig 6. The distribution observed for C22cmap shows surprisingly good agreement with C36 (particularly, C36s) force fields. From *P*(*R*_*g*_) we found 〈*R*_*g*_〉 = 15.8 ± 0.2 Å, which is almost equal to that observed for C36s (〈*R*_*g*_〉 = 15.9 Å), somewhat smaller than that of C36 (16.9Å), but larger than the C22* result (14.5Å). Taken together, C22cmap simulations exhibit a weak helix propensity in the C-terminal consistent with the formation of large number of stable short-range contacts that positions the C22cmap conformational ensemble in-between C36 and C22* ensembles (see Discussion for further analysis). The representative structure of A*β*10-40 peptide in C22cmap force field is displayed in Fig 1e.

### Conformational ensemble of A*β*10-40 monomer in OPLS-AA force field

To evaluate A*β*10-40 conformational ensemble in the force field unrelated to any CHARMM version, we have performed OPLS-AA REMD simulations. Similar to all previously considered force fields Fig 2 and Table 1 show that A*β*10-40 in OPLS-AA simulations adopt mainly turn (〈*T*〉 = 0.46 ± 0.02) or random coil (〈*RC*〉 = 0.40 ± 0.03) conformations, which together represent 86% of all amino acid states. However, in contrast to other force fields (e.g., C36) *β*-state fraction is elevated four-fold to 〈*S*〉 = 0.12 ± 0.02, whereas *α*-helix occurrence is negligible. Fig 3, which displays residue-specific secondary structure propensities, further underscores the characteristic OPLS-AA feature—an elevated sampling of *β*-structure in the S2 and S4 hydrophobic regions, where for some positions *i* 〈*S*(*i*)〉 reaches ≈0.4. The helix propensity across A*β* sequence is uniformly weak, whereas the turn and random coil fractions generally follow the other force field trends. Accordingly, the RMSD comparing the *β* distributions 〈*S*(*i*)〉 in OPLS-AA and C36 simulations has a large value of 0.13, whereas the RMSD for helix and turn propensities are much smaller (0.04 and 0.08). Fig 4e displays the distributions of dihedral angle RMSF, *δϕ*(*i*) and *δψ*(*i*), which are qualitatively similar to their C36 counterparts. In fact, the average 〈*δϕ*〉 = 54.4 ± 1.2° and 〈*δψ*〉 = 91.1 ± 1.8° are close to the C36 values. Consequently, the RMSD comparing *δϕ*(*i*) and *δψ*(*i*) distributions from OPLS-AA and C36 simulations are relatively small (20.2° and 12.8°). These findings indicate that the *β*-structure occurs in A*β* peptide transiently and does not strongly affect backbone fluctuations.

The equilibrium contact map 〈*C*(*i*, *j*)〉 displayed in Fig 5e clearly exhibits more extensive tertiary interactions forming in OPLS-AA comparing to C36 force field. Indeed, the numbers of all and long-range contacts are increased by about 50% and 100%, respectively, to 〈*C*〉 = 30.8 ± 0.5 and 〈*C*_*LR*_〉 = 21.1 ± 0.6 resulting in the largest fraction of long-range interactions of 69% among all force fields tested by us. Interestingly, even though OPLS-AA promotes tertiary interactions, very few of them qualify as stable. Specifically, in sharp contrast to C22* Table 6 lists only one stable long-range (Val18-Leu34) and one stable short-range (Tyr10-Val12) contacts. Among top five long-range contacts four are hydrophobic and three link the sequence regions S2 and S4 (Val18-Leu34, Val18-Val36, Leu17-Met35) with the probabilities of occurrence close to the stability threshold. Also, unique to OPLS-AA simulations, the salt-bridge Asp23-Lys28 appears among the top five long-range contacts. Finally, no long- or short-range top five contacts are shared between OPLS-AA and C36 simulations. It is then not surprising that extensive but weak tertiary interactions formed in OPLS-AA simulations lead to a fairly large RMSD value measuring the difference between the contact maps obtained in OPLS-AA and C36 force fields (0.07).

**Table 6:** Five top side chain contacts in A*β*10-40 peptide for OPLS-AA force field.

long-range			short-range		
Rank	contact	〈*C*(*i*, *j*)〉	Rank	contact	〈*C*(*i*, *j*)〉
1	18–34	0.35 ± 0.13	1	10–12	0.37 ± 0.02
2	18–36	0.34 ± 0.17	2	25–29	0.28 ± 0.14
3	19–24	0.33 ± 0.14	3	34–36	0.28 ± 0.05
4	23–28	0.31 ± 0.11	4	11–13	0.28 ± 0.02
5	17–35	0.30 ± 0.14	5	35–37	0.27 ± 0.09

Probability distribution *P*(*R*_*g*_) for A*β*10-40 radius of gyration presented in Fig 6 is far more narrow and reaches maximum at the smallest *R*_*g*_ compared to any other force field. We determined from Fig 6 that the equilibrium value 〈*R*_*g*_〉 is 13.5 ± 0.2Å, which is the smallest among the force fields tested. Similar observation holds for the end-to-end distance (〈*R*_1*N*_〉 = 15.6 ± 1.5 Å). Thus, OPLS-AA force field promotes extensive but flickering tertiary interactions resulting in A*β*10-40 collapse and moderate enhancement of *β*-structure as shown in Fig 1f.

### Comparison of experimental and computational J-coupling and RDC constants

**Fig 7:**
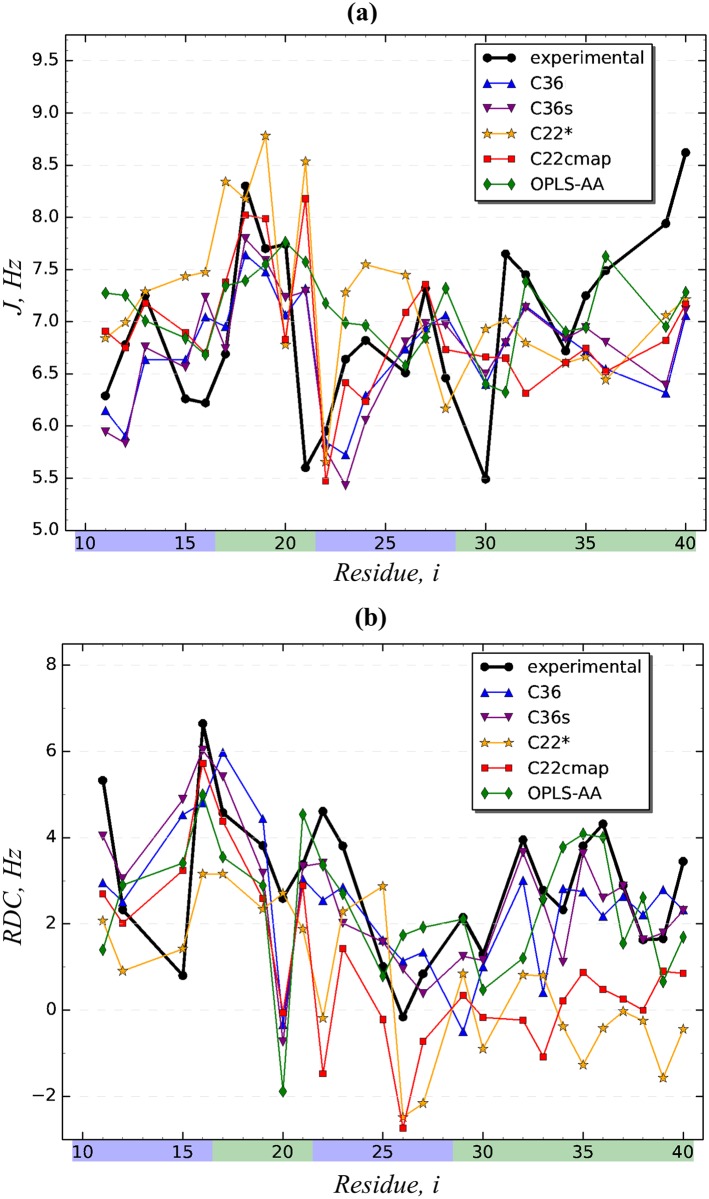
Comparison of *in silico* and experimental structural data. (a) Distributions of ^3^*J*_*HNHα*_-coupling constants, *J*_*comp*_(*i*), computed for A*β*10-40 peptide using Pardi *et al* coefficients for Karplus equation [51] and REMD sampling generated with five force fields. Superimposed are experimental ^3^*J*_*HNHα*_-coupling constants, *J*_*exp*_(*i*) (black lines with circles) measured by Roche *et al* [18]. This combination of Karplus equation coefficients and experimental data provides the best agreement between *J*_*comp*_(*i*) and *J*_*exp*_(*i*). Only amino acids with experimentally available data are considered. (b) Distributions of RDC constants computed for A*β*10-40 peptide using PALES program [55] and REMD sampling generated with five force fields. Superimposed are experimental RDC constants (black lines with circles) measured by Wang and coworkers [54].

We have compared ^3^*J*_*HNHα*_-coupling and RDC constants computed from our simulations and measured experimentally (see Materials and Methods and Fig 7a). (It is important to note that experimental measurements refer to the full-length peptide A*β*1-40, whereas our simulations have examined the amino-truncated fragment A*β*10-40. This point is elaborated in Discussion.) We first investigated the agreement between ^3^*J*_*HNHα*_-coupling constants. As stated in the Materials and Methods we used three sets of experimental ^3^*J*_*HNHα*_-coupling constants, *J*_*exp*_, measured by Garcia and coworkers [16, 17] and, more recently, by Bax and coworkers [18]. Furthermore, to compute ^3^*J*_*HNHα*_-coupling constants *in silico*, *J*_*comp*_, we applied Eq (1) with three different sets of Karplus equation coefficients [51–53]. Because *a priori* it is unclear which combination of experimental data and Karplus equation coefficients is more accurate, we considered all nine possible combinations and for each computed *J*_*comp*_(*i*)-coupling constants using A*β*10-40 sequence positions *i* with available experimental measurements. Consistency between *J*_*comp*_(*i*) and *J*_*exp*_(*i*) was evaluated by calculating the root-mean-square deviation (RMSD), quality function *Q*, and Pearson’s correlation coefficient (PCC) as described in the Materials and Methods. These quantities were then averaged over nine datasets and are presented in Table 7. Judged by RMSD, PCC, and *Q* values as well as their errors the best agreement between experimental and computational *J*-coupling constants is observed for C36 and C36s force fields. The ranking of other force fields in the descending order of agreement with the experiment is OPLS-AA, C22cmap, and C22*. Notably, C22* shows very significant increase in RMSD (by 41%) compared to C36 and has effectively no correlation with *J*_*exp*_(*i*) as measured by PCC (0.14).

**Table 7:** Comparison of experimental and computational *J*-coupling and RDC constants^†^.

Force Field	Water Model	^3^*J*_*HNHα*_ (*Hz*)			RDC (*Hz*)		
		RMSD	PCC	Q	RMSD	PCC	Q
CHARMM36	mTIP3P	0.88±0.07	0.42±0.06	0.13±0.01	1.63±0.09	0.48±0.08	0.49±0.03
CHARMM36	sTIP3P	0.89±0.10	0.44±0.09	0.13±0.01	1.36±0.27	0.65±0.08	0.41±0.08
CHARMM22*	mTIP3P	1.24±0.04	0.14±0.02	0.18±0.01	2.80±0.15	0.49±0.07	0.85±0.05
CHARMM22/cmap	mTIP3P	1.03±0.06	0.24±0.06	0.15±0.01	2.57±0.08	0.57±0.03	0.78±0.02
OPLS-AA	mTIP3P	0.95±0.02	0.38±0.06	0.14±0.00	1.75±0.10	0.42±0.06	0.53±0.03

^†^^3^*J*_*HNHα*_ related values are averages over nine combinations of experimental data/Karplus equation parameters

To provide additional probe of A*β*10-40 conformational ensemble we have computed the RDC constants, *RDC*_*comp*_(*i*), as described in the Materials and Methods and S1 Text. To compare them with their experimental counterparts, *RDC*_*exp*_(*i*), [54] shown in Fig 7b, we used the same metrics as for ^3^*J*_*HNHα*_-coupling constants. The results presented in Table 7 indicate that the best agreement between experimental and computational RDC constants is observed for C36s force field, which has the smallest RMSD or *Q* and the largest PCC of 0.65. Compared to C36s force field, C36 exhibits larger RMSD and Q (by ≈ 20% for both) and significantly lower PCC (a 25% decrease). With respect to RMSD and Q the worst agreement is seen for C22cmap and C22*, for which the RMSD or *Q* values are about two-fold larger than for C36s. Their PCC values are also lower than for C36s, but higher, especially of C22cmap, than for C36. Measured by RMSD and *Q* the agreement with the experiment places OPLS-AA between C36 and C22cmap/C22*. However, OPLS-AA demonstrates the worst correlation between computational and experimental RDC constants as measured by PCC. Thus, taken together our data suggest that the force field providing the best consistency with the experimental data is C36s. If we disregard a negligible (within the error) difference in *J*-coupling RMSD for C36s and C36 (Table 7), this conclusion is supported by all comparison metrics. With the exception of RDC PCC, other metrics identify C36 as the next “best” force field. Again, with the exception of PCC for RDC all the metrics determine that C22* produces the worst agreement with the experimental data. The implications of our findings for the selection of force field and water model and for the differences between A*β*1-40 and A*β*10-40 conformational ensembles are presented in the Discussion.

## Discussion

### Comparison of A*β*10-40 conformational ensembles in different force fields

Using REMD we have performed a comparative analysis of A*β*10-40 conformational ensembles generated by employing five force fields, which combine four protein parameterizations (C36, C22*, C22cmap, and OPLS-AA) and two water models (standard and modified TIP3P). As a reference in our analysis we took the recent modification of CHARMM force field, CHARMM36, coupled with modified TIP3P water model. Its selection was motivated by recent tests showing that this force field provides the best agreement with experimental NMR data collected for six proteins [57]. Taken together, our results suggest several observations. First, all force fields produce fairly consistent distributions of secondary structure. According to Fig 2 and Table 1 all of them predict largely similar fractions of turn conformations (varying between 0.44 and 0.52) and, to a lesser extent, random coil (varying between 0.30 and 0.49). In all force fields, the turn structure dominates within approximately the same sequence regions, His13 and His14 in S1 region and a sequence interval Phe19-Gly29, whereas the random coil occurs at A*β*10-40 termini. Nevertheless, there are also significant variations among the force fields. For example, a unique feature of C22* force field is a considerable helix bias in the S3 and S4 regions resulting in 〈*H*(*i*)〉 ≈ 0.4 for few positions. Similarly, OPLS-AA differs from other simulations by significant *β*-structure propensity in the S2 and S4 regions, where 〈*S*(*i*)〉 peaks at ≈ 0.4.

Second, additional insight into peptide conformational ensemble is provided by the fluctuations in backbone dihedral angles, *δϕ*(*i*) and *δψ*(*i*). Similar to secondary structure, backbone fluctuations in C36 and C36s are in excellent agreement as evidenced by their consistent average values and small RMSDs. C22cmap differs moderately from C36 by having slightly more rigid backbone (i.e., smaller average 〈*δϕ*〉 and 〈*δψ*〉 values), whereas OPLS-AA, in contrast, demonstrates enhanced backbone fluctuations. However, the force field clearly standing apart from others is C22*, which predicts suppressed fluctuations in two sequence regions (Val17-Asp23 and Leu34-Val36). This feature plus generally small fluctuations in *ϕ* angles result in the lowest averages 〈*δϕ*〉 and 〈*δψ*〉 as well as elevated, by an order of magnitude, RMSD value between C22* and C36 simulations as opposed to that between C36 and C36s. Therefore, when secondary structure and backbone fluctuations are considered together, the force fields can be ranked in the descending order of similarity to C36 as C36s, which is nearly identical to C36, C22cmap and OPLS-AA, which moderately differ from C36, and C22*, which reveals considerably differences due to helix formation and rigid backbone.

It is important to consider our results in the context of other studies of force field propensities. C22cmap tendency to bias peptide conformational ensembles toward helical states has been documented for Ala pentamer [58], to correct which C36 force field has been developed [39]. Our results show that C22cmap indeed produces a slightly elevated helix fraction in unstructured A*β*10-40 compared to C36 or C36s increasing it to 0.12 from 0.04 or 0.06, respectively. However, this bias is weak compared to that of C22*, which demonstrates much stronger helix propensity in the C-terminal. Small differences in helix propensities between C22cmap and C36 have also been noted for the unstructured fragments of NTL9 peptides [31].

Our third observation is related to the distribution of tertiary interactions. In A*β* peptide C36 force field produces no stable long-range interactions and only two stable short-range contacts and, overall, it leads to the smallest numbers of tertiary contacts (21.1) and long-range contacts (10.7). A*β* conformations in this force field are also the least compact being characterized by the largest radius of gyration (16.9 Å). Thus, we conclude that equilibrium C36 conformations are dominated by expanded structures lacking stable interactions. C36s force field, which differs from C36 solely by the water model, generates very similar conformational ensemble, which also lacks stable interactions and shares four or three common top long- and short-range contacts with C36. Overall, the contact map RMSD for C36 and C36s is very low (0.02). Nevertheless, the C36s numbers of contacts, including long-range, are slightly larger (by 10–20%) than in C36. Additionally, compared to C36 C36s features slighly smaller A*β* radius of gyration. These results are consistent with recent comparison of standard and modified TIP3P water models, which showed that the latter enhances hydration and generates more open peptide conformations [32].

Although A*β* structures produced with C22cmap and C22* share some similarity (three long- and short-range top contacts are common), C22* is by far unique in generating the largest number of stable long-range contacts (eight as opposed to one in C22cmap). Some of these contacts, such as salt-bridge Lys16-Asp23, are effectively always formed. A distinctive characteristic of C22cmap is the large number (9) of stable short-range contacts combined with very few (1) stable long-range interactions. According to contact map RMSD, C22cmap differs moderately from C36 (0.07), whereas C22* deviates from C36 by far larger degree (0.12). There are no common top long- or short-range contacts between C36 and C22cmap or C22*. The tertiary interactions also set OPLS-AA force field apart from all other simulations. OPLS-AA generates the largest number of all contacts and, more importantly, the largest number of long-range contacts, which is increased almost two-fold compared to other simulations (except for C22*, with respect to which 〈 *C*_*LR*_ 〉 increases one-third). As a result OPLS-AA conformations exhibit extremely large fraction of long-range interactions reaching almost 70% and, accordingly, A*β* adopts most compact structures (〈*R*_*g*_〉 13.5 Å). Similar observation concerning A*β*1-40 peptide collapse has been made previously for OPLS-AA/L force field [30]. Interestingly, OPLS-AA tertiary contacts, although numerous, are weak suggesting that A*β* samples compact but still disordered states. OPLS-AA and C36 do not share common top interactions, whereas the contact map RMSD between the two is moderate (0.07). Thus, using contact map RMSD and C36 as reference we rank the force fields in the descending order of similarity as C36s, C22cmap, OPLS-AA (due to compact state), and C22*.

It might be argued that the computed A*β*10-40 conformational ensembles are specific to 330K. To investigate this possibility we used our REMD sampling to recompute the secondary structure propensities for five force fields at 300K. Fig D and Table A in S1 Text represent the analogues of Fig 2 and Table 1 obtained at 300K. It is seen that the secondary structure propensities at 300K and 330K are qualitatively similar, differring by no more than 7% for the propensities > 0.1. The same conclusion applies to the comparisons of the distributions of secondary structure along A*β*10-40 sequence, which demonstrate, for instance, that C22* helical propensity < *H*(*i*) > only slighly increases at 300K. Finally, decrease in temperature triggers no qualitative changes in tertiary interactions (see S1 Text). Therefore, we surmize that A*β*10-40 conformational ensembles at 300K and 330K appear qualitatively similar.

Combining the analysis of secondary and tertiary structures we make the following conclusions:

- All force fields predict that A*β*10-40 adopts unfolded structure dominated by turn and random coil conformations;
- Water model does not dramatically affect secondary or tertiary A*β* peptide structure with standard TIP3P model favoring slightly more compact states;
- Although there are no significant differences in secondary structures observed in C36 and C22cmap simulations, little similarity is seen in their tertiary interactions.
- Unique features of OPLS-AA force field are moderate *β*-structure propensity and extensive, but flickering long-range tertiary interactions leading to A*β* collapse. There are no common top tertiary interactions between C36 and OPLS-AA force fields.
- Unique features of C22* are moderate helix propensity and multiple, exceptionally stable long- and short-range interactions. Based on RMSD computations applied to secondary (helix, turn, and backbone fluctuations distributions) and tertiary (contact maps) structure, we conclude that this force field differs the most from C36.

### Which force field describes A*β*10-40 most accurately?

Using REMD simulations we have computed the ^3^*J*_*HNHα*_-coupling and RDC constants for the amino-truncated peptide A*β*10-40 and compared them to the experimental measurements performed for the full-length peptide A*β*1-40. Although A*β*1-40 and A*β*10-40 peptides are not entirely identical having 78% of sequence homology, their comparison can still be instructive as demonstrated below. We first evaluate the agreement between *in silico* A*β*10-40 and experimental A*β*1-40 data in light of similar assessments made in the literature for A*β*1-40 or A*β*1-42 peptides, in which identical peptide species were used both in the experiments and simulations [17, 27]. According to our analysis (Table 7) RMSD and PCC between the *in silico* and experimental *J*-coupling constants for the “best” C36s force field is 0.89 *Hz* and 0.44, respectively. Similar comparison made for A*β*1-42 using AMBERff99SB force field and TIP4P-Ew water model [27] yielded the RMSD values of 0.96 *Hz* or 1.46 *Hz* depending on the specific set of Karplus equation coefficients [52, 53] (the average is 1.21). The PCC values varied in the interval 0.4–0.5. If we restrict our computations of the average RMSD and PCC to the two coefficient sets used by Sgourakis *et al* [27], we obtain RMSD = 0.92 *Hz* and PCC = 0.44. (Note that previous studies have often performed fitting of Karplus equation coefficients to better represent experimental data [17, 27]. However, we opted against this adjustment to provide more unbiased assessment of force fields.) Our comparison of A*β*10-40 RDC constants produced with C36s to their A*β*1-40 experimental counterparts results in RMSD = 1.36 *Hz* and PCC = 0.65. Analogous comparisons made for A*β*1-42 led to RMSD = 1.49 *Hz* and 0.35 ≲ PCC ≲ 0.45 [27].

A recent study has compared *in silico* and experimental *J*-coupling and RDC constants for three peptides, A*β*1-42, A*β*1-40, and A*β*1-42-M35ox using OPLS-AA/L force field and TIP3P water model [17]. Using the coefficients of Vuister and Bax [53], the RMSD values for *J*-coupling distributions were 1.21, 1.29, and 1.06 *Hz*, respectively (the average is 1.19 *Hz*), whereas the PCC values were 0.50, 0.76, 0.49 (the average is 0.58). If we again restrict our computations of the RMSD and PCC to the Vuister and Bax coefficients, we find RMSD = 0.85 *Hz* and the PCC = 0.48. The comparison of the *in silico* and experimental RDC distributions for A*β*1-42, A*β*1-40, and A*β*1-42-M35ox [27] yielded the RMSD values of 1.66, 1.69, and 1.45 *Hz* (the average is 1.6 *Hz*), and the PCC values of 0.39, 0.50, and 0.44 (the average is 0.44). Finally, the third study has performed similar comparison of *in silico* and experimental J-coupling and RDC data using AMBERff99SB force field [28]. The RMSD values for J-coupling distributions were 0.99*Hz* for both A*β*1-40 and A*β*1-42. The RMSD values comparing RDC distributions were about 2.2*Hz* for both peptides.

Thus, if we consider the RMSD values comparing A*β*1-40 and A*β*10-40 peptides against the RMSD comparisons made previously for identical peptides, it becomes clear that the difference in *J*-coupling constants between A*β*1-40 and A*β*10-40 is actually smaller than the reported values for A*β*1-42, A*β*1-40, or A*β*1-42-M35ox (our RMSDs of 0.89, 0.92, or 0.85 *Hz* vs “their” RMSDs of 1.21, 1.19, or 0.99 *Hz*). The values of PCC calculated by us are about the same or slightly lower than those reported for the three peptides (our PCCs of 0.44, 0.44 or 0.48 vs “their” PCC in the range of 0.4–0.6). As shown above these conclusions hold irrespective of computing the RMSD and PCC using all Karplus equation coefficient sets or only specific sets. Similarly, as measured by RMSD the agreement between *in silico* A*β*10-40 and experimental A*β*1-40 RDC data is much better than the previous comparisons, which involved identical *in silico* and experimental peptide species, such as A*β*1-42, A*β*1-40, or A*β*1-42-M35ox (our RMSD of 1.36 vs “their” RMSD of 1.49, 1.6, or 2.2 *Hz*). The same conclusion is supported by PCC comparing RDC distributions, which is 0.65 in our study against the approximate range of 0.35 to 0.45 reported previously.

Taken together the analysis above suggests two conclusions. First, if *in silico* A*β*10-40 and experimental A*β*1-40 *J*-coupling and RDC constants are generally in better agreement than these quantities computed and measured for strictly identical peptides, then the differences in the conformational ensembles of A*β*10-40 and A*β*1-40 are likely to be small or, at least, not exceeding the force field errors in reproducing the conformations of a specific peptide (A*β*1-42, A*β*1-40, or A*β*1-42-M35ox). Therefore, guided by the previous validations of protein force fields against A*β* NMR data [16, 17, 27, 28] we argue that our analysis supports using A*β*10-40 peptide as a proxy of the full-length A*β*1-40. This conjecture has been made earlier by our [33, 59] and other [60] groups. In this context, we note that the OPLS-AA/L simulations of the full-length A*β*1-40 have predicted stable *β*-structure in Leu17-Ala21 and Ile31-Val36 regions [30]. In line with our view of A*β*10-40 as a proxy of A*β*1-40, the elevated *β*-structure propensity is observed in the same A*β*10-40 regions when sampled in our OPLS-AA simulations.

Second, a good agreement between *in silico* A*β*10-40 and experimental A*β*1-40 *J*-coupling and RDC constants argues that CHARMM36 force field with standard TIP3P water model is possibly the best force field for reproducing A*β* conformational ensemble. Previous studies evaluating the force fields for their ability to reproduce A*β* experimental data have identified OPLS-AA with TIP3P water model as most accurate [16]. However, to our knowledge CHARMM force fields have never been directly evaluated against the distributions of A*β* *J*-coupling and RDC constants. In this study we addressed this issue. Recently, eight different force fields were evaluated using REMD simulations for their ability to reproduce small-angle X-ray scattering and NMR data for five natively unstructured peptides [61]. The authors have determined that CHARMM22* generates the conformational ensembles most consistent with the experiments. They also noted erroneous CHARMM36 propensity to sample left-handed *α*-helix conformations. However, our study did not reach the same conclusions for A*β* peptides suggesting that the selection of the “best” force field still depends on the peptide and details of simulations. Incidentally, an updated version of CHARMM36 force field has been recently released, which corrects left-handed *α*-helix bias [62]. However, in the case of A*β*10-40 peptides this modification appears as not critically necessary given the lack of A*β* left-handed *α*-helix in the original CHARMM36 force field.

### Conclusion

By applying REMD simulations we have performed comparative analysis of the conformational ensembles of amino-truncated A*β*10-40 peptide produced with five force fields, which combine four protein parameterizations (CHARMM36, CHARMM22*, CHARMM22/cmap, and OPLS-AA) and two water models (standard and modified TIP3P). A*β*10-40 conformations were characterized by the analysis of secondary structure, backbone fluctuations, tertiary interactions, and radius of gyration. In addition, using computed conformational ensembles we have calculated A*β*10-40 ^3^*J*_*HNHα*_-coupling and RDC constants and compared them with their experimental counterparts obtained for the full-length A*β*1-40 peptide. Taken together, our study led us to several conclusions. First, all force fields predict that A*β* adopts unfolded structure dominated by turn and random coil conformations. Second, specific TIP3P water model does not dramatically affect secondary or tertiary A*β*10-40 peptide structure, albeit standard TIP3P model favors slightly more compact states. Third, although the secondary structures observed in CHARMM36 and CHARMM22/cmap simulations are qualitatively similar, their tertiary interactions show little consistency. Fourth, two force fields have unique features setting them apart from CHARMM36 or CHARMM22/cmap. Specifically, OPLS-AA reveals moderate *β*-structure propensity coupled with extensive, but weak long-range tertiary interactions leading to A*β* collapse. CHARMM22* exhibits moderate helix propensity and generates multiple, exceptionally stable long- and short-range interactions. There are no common frequent tertiary interactions between CHARMM36 and OPLS-AA or CHARMM22* force fields. Our investigation suggests that among all force fields CHARMM22* differs the most from CHARMM36. Fifth, the analysis of ^3^*J*_*HNHα*_-coupling and RDC constants based on CHARMM36 force field with standard TIP3P model led us to an unexpected finding that *in silico* A*β*10-40 and experimental A*β*1-40 constants are generally in better agreement than these quantities computed and measured for identical (100% homologous) peptides, such as A*β*1-40 or A*β*1-42. On the basis of this observation we argued that the differences in the conformational ensembles of A*β*10-40 and A*β*1-40 are likely to be small and the former can be used as proxy of the full-length peptide. We also concluded that CHARMM36 force field with standard TIP3P model produces the most accurate representation of A*β*10-40 conformational ensemble.

## Supporting Information

S1 Text Supporting information providing additional details and data. (PDF)
