# Supplementary material for: Is the Conformational Ensemble of Alzheimer’s Aβ10-40 Peptide Force Field Dependent?
Source: PLoS Comput Biol. 2017 Jan 13;13(1):e1005314. doi: 10.1371/journal.pcbi.1005314 (PMC5279813; doi:10.1371/journal.pcbi.1005314)
Supplement: S1 Text — (PDF) [file pcbi.1005314.s001.pdf]

C. M. Siwy, C. Lockhart, and D. K. Klimov "Is the Conformational Ensemble of Alzheimer's Aβ10-40 Peptide Force Field Dependent?"

Supporting Information

Convergence of REMD simulations: We performed replica exchange molecular dynamics (REMD) simulations of the Aβ10-40 peptide in water using five different force fields, including CHARMM36 with modified TIP3P water model (C36), CHARMM36 with standard TIP3P water model (C36s), CHARMM22* with modified TIP3P water model (C22*), CHARMM22 with CMAP corrections and modified TIP3P water model (C22cmap), and OPLS-AA with modified TIP3P water model (OPLS-AA). As an example we present the analysis of simulation convergence for the system studied using the CHARMM36 force field with modified TIP3P water model, which provides one of the best agreement with experimental measurements in our study. Simulation convergence is assessed through several quantities. First, we computed the number N_s of unique states (E_p, C) sampled at least once during simulations, where E_p is the potential energy of the entire system and C is the number of intrapeptide contacts. In the definition of the unique states (E_p, C), the potential energy E_p, which ranged in the interval from -48912.4 to -41503.8 kcal/mol, was binned using the interval of 1 kcal/mol. Fig. A shows N_s plotted over the cumulative equilibrium simulation time τ_{sim}. Saturation of N_s at $\tau_{sim} \approx$ 0.8 μs indicates an approximate depletion in unexplored conformational states. This observation is taken as a necessary condition for simulation convergence. Similar $N_s(\tau_{sim})$ dependencies have been observed for other force fields. It should be added that slow residual growth of N_s by the end of REMD simulations does not affect the quality of ensemble averages. For example, restricting the data analysis to the last 1/3 of sampled states does not change the conclusions about the quality of force fields (for example, with respect to the analysis of J-coupling and RDC constants reported in Table 7).

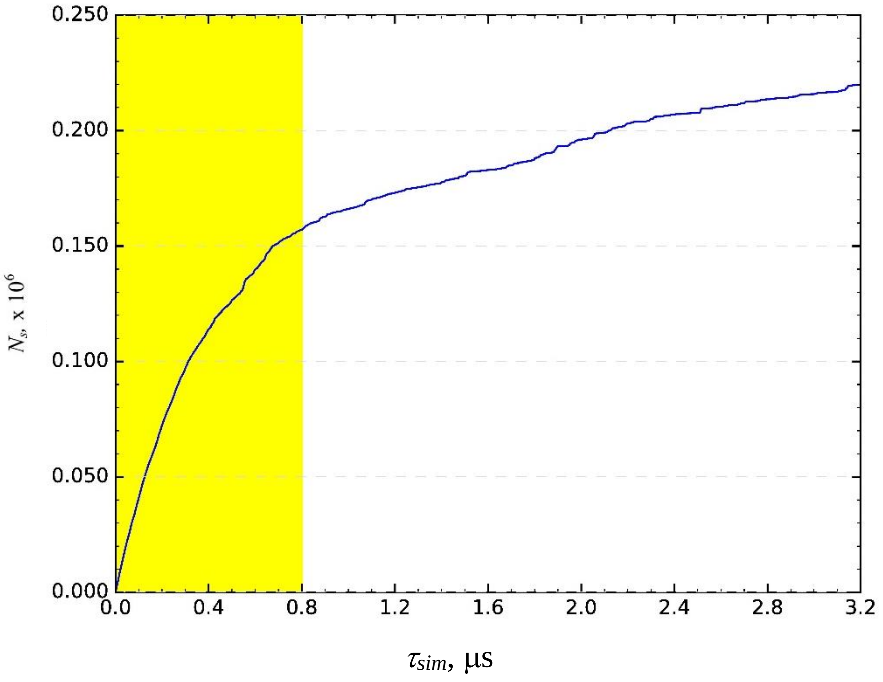

Fig. A The number N_s of unique states (E_p, C) as a function of the cumulative equilibrium simulation time τ_{sim} collected in all replicas The yellow region marks the initial acquisition of new states before approximate N_s saturation.

Second, we demonstrate REMD performance by plotting in Fig. B the random walk of replicas over temperatures during a typical trajectory. Appearance of a color mosaic indicates a rapid mixing of replicas over temperatures. Importantly, the lack of fixed colors favoring any particular temperature indicates that replicas are able to avoid becoming trapped in local free energy minima.

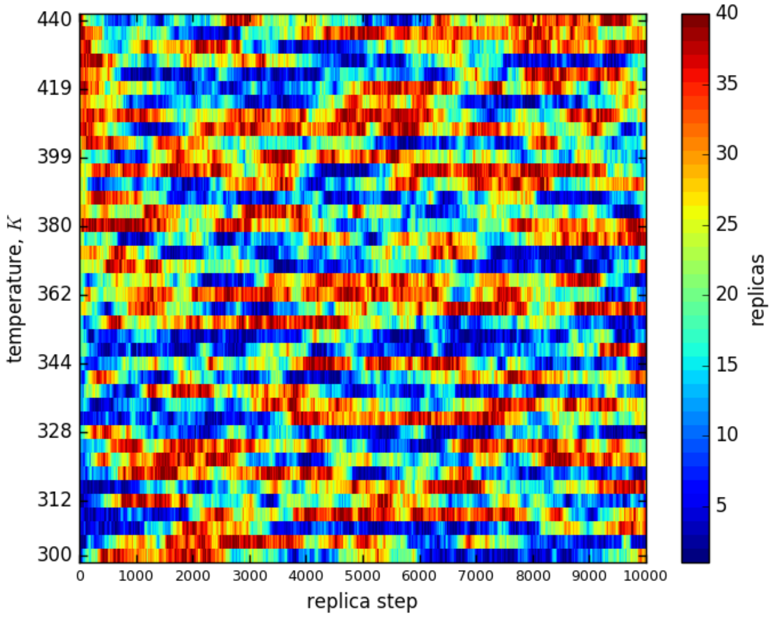

Fig. B Random walk of replicas over temperatures in a typical REMD trajectory. Colors in the right scale represent the distribution of replicas over temperatures at the beginning of the trajectory.

Third, we computed the replica mixing parameter *m(T)* introduced by Han and Hansmann [1]. This parameter is defined as

$$m(T) = 1 - \frac{\sqrt{\sum_{r=1}^{R} t_r^2}}{\sum_{r=1}^{R} t_r},$$

where t_r is the amount of time spent by the replica r at the temperature T. If all R replicas are equally represented at all temperatures sampled in REMD, then $m(T) = 1-1/\sqrt{R}$, which is the optimal theoretical value independent of temperature. When $R = 40$, the optimal value of *m(T)* is therefore 0.84. Importantly, *m(T)* allows us to quantitatively assess the distribution of replicas over temperatures in all REMD trajectories taken together. In Fig. C, we plot *m(T)* for all four trajectories of the Aβ10-40 peptide in water simulated using C36, C36s, C22*, and OPLS-AA force fields. Approach of *m(T)* to an

optimal theoretical value at all temperatures *T* for all force fields suggests nearly ideal replica mixing over temperatures.

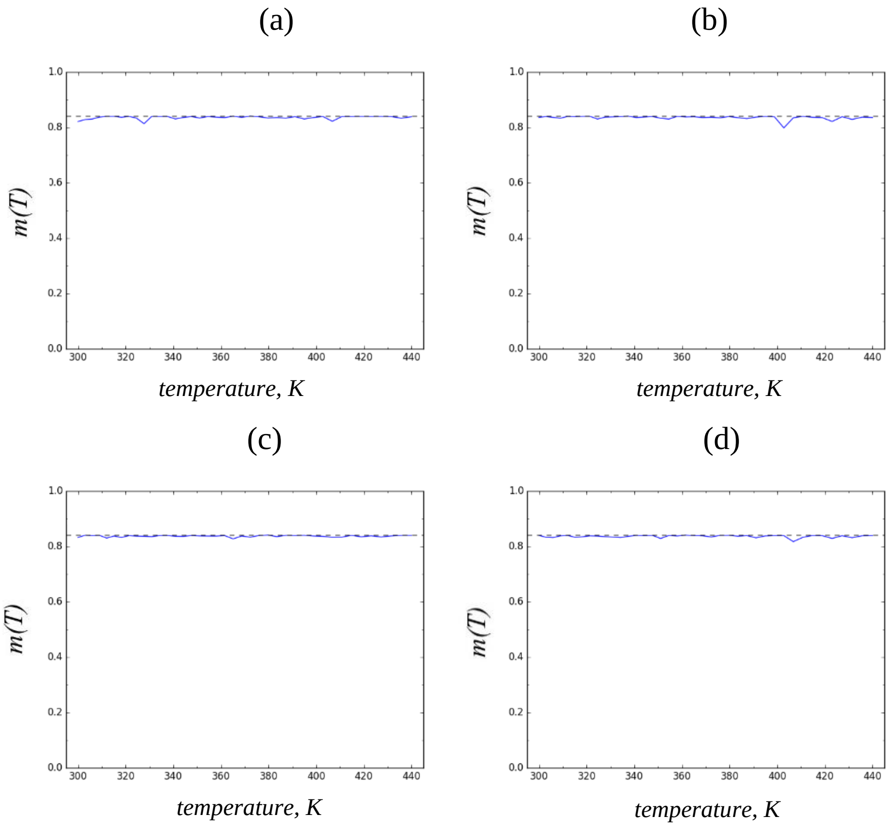

Fig. C The replica mixing parameter *m(T)* computed as a function of REMD temperatures *T* for all four REMD trajectories combined. Panels (a)-(d) present *m(T)* for C36, C36s, C22*, and OPLS-AA force fields, respectively. The dashed line represents the optimal theoretical value of *m(T)* = 0.84.

Temperature dependence of Aβ10-40 conformational ensemble: To investigate the impact of temperature we have recomputed Aβ10-40 secondary structure propensities in five force fields at 300K using REMD sampling. The overall fractions of helix, turn, random coil, and β secondary structures for each of the five force fields are displayed in Fig. D and listed in Table A. Similar to the data at 330K in Fig. 2 and Table 1, turn and random coil conformations remain dominant at 300K in all the force fields. Furthermore, as at 330K C22* and OPLS-AA show moderate helix and β-structure propensities. To evaluate tertiary structure, we have recomputed at 300K the numbers of all and long-range side chain contacts, $\langle C \rangle$ and $\langle C_{LR} \rangle$, in five force fields. We found only a minor increase in $\langle C \rangle$ and $\langle C_{LR} \rangle$ (the largest is observed for OPLS-AA, for which $\langle C \rangle = 31.2 \pm 0.4$ and $\langle C_{LR} \rangle = 21.5 \pm 0.5$). Importantly, no changes in ranking order or in the distribution of stable interactions were observed compared to 330K data.

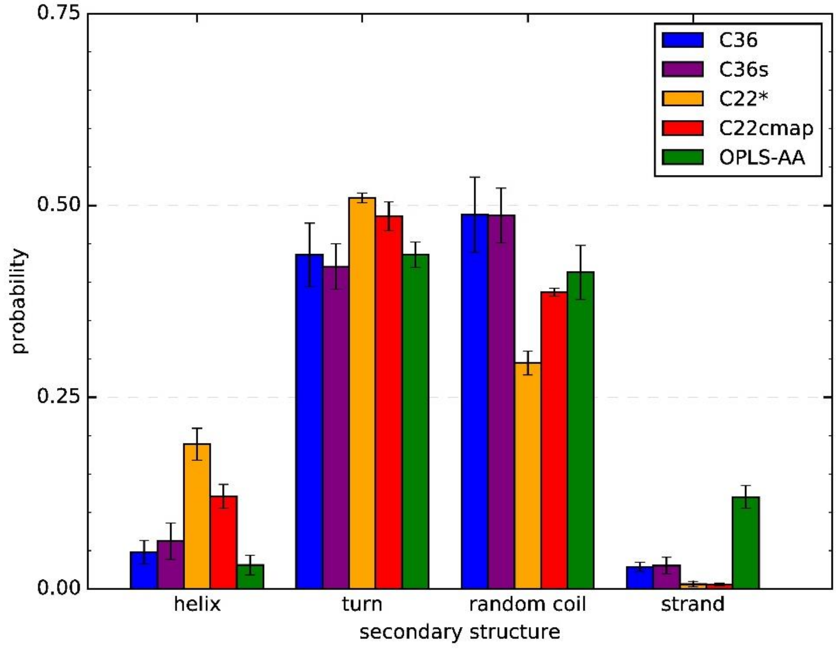

Fig. D Fractions of Aβ10-40 secondary structure for five force fields computed at 300K.

Table A Secondary structure in Aβ10-40 peptide at 300K.

**Force Field**	**Water Model**	**<H>**	**<T>**	**<RC>**	**<S>**
CHARMM36	mTIP3P	0.05±0.02	0.44±0.04	0.49±0.05	0.03±0.01
CHARMM36	sTIP3P	0.06±0.02	0.42±0.03	0.49±0.04	0.03±0.01
CHARMM22*	mTIP3P	0.19±0.02	0.51±0.01	0.29±0.02	0.01±0.00
CHARMM22/cmap	mTIP3P	0.12±0.02	0.49±0.02	0.39±0.00	0.01±0.00
OPLS-AA	mTIP3P	0.03±0.01	0.44±0.02	0.41±0.04	0.12±0.01

Selection of alignment option for RDC computations: Computations of RDC constants were performed using global alignment of Aβ10-40 conformations. Although we considered using local alignment option, we have opted against it for three reasons. First, previous study has shown that, if large conformational sample of disordered protein is utilized (>10^4), in silico RDC constants computed via global or local alignments correlate with the experimental data equally well [2]. Because for each force field we have produced 1.6×10^6 conformations, this condition is met in our simulations. Second, previous REMD simulations have directly compared the RDC values predicted via global and local alignments for AMBERff99SB force field [3]. The local alignment was found to slightly reduce the RMSD values comparing in silico and experimental distributions. However, even the RMSD values corresponding to local alignment were still larger (>1.88) than those computed by us for the three “best” force fields in Table 7 employing global alignment option. In addition, we performed the following test. Fig. 7 shows large systemic deviations of RDC constants computed using C22cmap from experimental values in Aβ C-terminal. We have recomputed RDC constants restricting the alignment

to the C-terminal, but did not observe an improvement in the agreement between in silico and experimental distributions. For these reasons, we have selected global alignment of peptide structures for computing RDC constants.

Structural database: To facilitate further analysis of Aβ10-40 conformations, we have created a depository of Aβ10-40 structures collected at 330K using the five force fields, C36, C36s, C22*, C22cmap, and OPLS-AA. This structural database is available at binf.gmu.edu/dklimov/Abeta10-40/Ab1040Data.tar.gz.

References

[1] Han, M. and Hansmann, U.H.E. (2011) Replica exchange molecular dynamics of the thermodynamics of fibril growth of Alzheimer's Aβ42 peptide. *J. Chem. Phys.* **135**, 065101.

[2] Marsh, J. A., Baker, J. M. R., Tollinger, M., and Forman-Kay, J. D. (2008) Calculation of residual dipolar couplings from disordered state ensembles using local alignment. *J. Amer. Chem. Soc.* **130**, 7804-7805.

[3] Ball, K. A., Phillips, A. H., Wemmer, D. E., and Head-Gordon, T. (2013) Differences in β-strand populations of monomeric Aβ40 and Aβ42. *Biophys. J.* **104**, 2714-2724.
